# Role of Hepatic Glucocorticoid Receptor in Metabolism in Models of 5*α*R1 Deficiency in Male Mice

**DOI:** 10.1210/en.2019-00236

**Published:** 2019-06-14

**Authors:** Tracy C S Mak, Dawn E W Livingstone, Mark Nixon, Brian R Walker, Ruth Andrew

**Affiliations:** University/British Heart Foundation Centre for Cardiovascular Science, Queen’s Medical Research Institute, University of Edinburgh, Edinburgh, United Kingdom

## Abstract

Inhibition of 5*α*-reductases impairs androgen and glucocorticoid metabolism and induces insulin resistance in humans and rodents. The contribution of hepatic glucocorticoids to these adverse metabolic changes was assessed using a liver-selective glucocorticoid receptor (GR) antagonist, A-348441. Mice lacking 5*α*-reductase 1 (5*α*R1-KO) and their littermate controls were studied during consumption of a high-fat diet, with or without A-348441(120 mg/kg/d). Male C57BL/6 mice (age, 12 weeks) receiving dutasteride (1.8 mg/kg/d)) or vehicle with consumption of a high-fat diet, with or without A-348441, were also studied. In the 5*α*R1-KO mice, hepatic GR antagonism improved diet-induced insulin resistance but not more than that of the controls. Liver steatosis was not affected by hepatic GR antagonism in either 5*α*R1KO mice or littermate controls. In a second model of 5*α*-reductase inhibition using dutasteride and hepatic GR antagonism with A-348441 attenuated the excess weight gain resulting from dutasteride (mean ± SEM, 7.03 ± 0.5 vs 2.13 ± 0.4 g; dutasteride vs dutasteride plus A-348441; *P* < 0.05) and normalized the associated hyperinsulinemia after glucose challenge (area under the curve, 235.9 ± 17 vs 329.3 ± 16 vs 198.4 ± 25 ng/mL/min; high fat vs high fat plus dutasteride vs high fat plus dutasteride plus A-348441, respectively; *P* < 0.05). However, A-348441 again did not reverse dutasteride-induced liver steatosis. Thus, overall hepatic GR antagonism improved the insulin resistance but not the steatosis induced by a high-fat diet. Moreover, it attenuated the excessive insulin resistance caused by pharmacological inhibition of 5*α*-reductases but not genetic disruption of 5*α*R1. The use of dutasteride might increase the risk of type 2 diabetes mellitus and reduced exposure to glucocorticoids might be beneficial.

The consequences of glucocorticoid excess have been well established, with links to metabolic disease exemplified by patients with Cushing syndrome. Glucocorticoids primarily mediate their actions through the glucocorticoid receptor (GR), activation of which can be regulated by prereceptor metabolism within tissues. For example, 11*β*-hydroxysteroid dehydrogenase types 1 and 2 have been shown to modulate GR and mineralocorticoid receptor activation, respectively (1, 2). The potential role of other enzymes such as the 5*α*-reductases (5*α*Rs), which are responsible for the rate-limiting step in glucocorticoid metabolism in modifying GR activation, has been less clearly established.

Two main isozymes of 5*α*Rs can metabolize many pregnene steroids. Type 2 5*α*R (5*α*R2) is highly expressed in the male reproductive tract, where it amplifies androgen action. In contrast, 5*α*R type 1 (5*α*R1) is most highly expressed in the liver but also in adipose tissue and skeletal muscle at lower levels (3, 4). 5*α*R inhibitors such as finasteride (which inhibits type 2) and dutasteride (which inhibits both isozymes) are prescribed for many men with prostate disease (5). A recent population-based cohort study found the risk of developing new-onset type 2 diabetes was greater in men exposed to 5*α*R inhibitors compared with those receiving tamsulosin (6). Furthermore, human studies have shown that the combined inhibition of 5*α*R1 and 5*α*R2 (but not 5*α*R2 alone) impaired insulin sensitivity and resulted in increased hepatic fat content compared with measurements before dutasteride treatment (7, 8). Similarly, genetic disruption of 5*α*R1, but not 5*α*R2, in 5*α*R1 knockout (5*α*R1KO) mice also caused hyperinsulinemia, weight gain, increased hepatic triglyceride deposition, and a predisposition toward irreversible liver disease (4, 9).

Given the abundance of 5*α*R1 in the liver and the characterized hepatic phenotype in 5*α*R1KO mice and humans receiving 5*α*R inhibitors, it is likely that the liver is a crucial site of steroid accumulation after 5*α*R1 inhibition or deficiency. Although accumulation of either androgens or glucocorticoids could be responsible for the adverse metabolic effects, androgen accumulation appears a less likely mechanism. Nonselective inhibition of 5*α*Rs in obese Zucker rats induced fatty liver in both castrated and intact rats (4), and female 5*α*R1KO mice were more susceptible to hyperinsulinemia than were males (10). Therefore, we hypothesized that hepatic glucocorticoid excess underpins the adverse metabolic changes observed after 5*α*R1 inhibition or disruption, driving hepatic steatosis and insulin resistance.

Systemic GR antagonism with compounds such as mifepristone (RU486) improves insulin and glucose homeostasis in *ob/ob* mice (11); however, the site of action is uncertain and likely multiple, with effects confounded by compensatory hypercorticosteronemia. To test our hypothesis that the metabolic phenotype in mice with disruption of 5*α*R1 is driven by excess glucocorticoid action in the liver, we used the liver-selective GR antagonist, A-348441. A-348441 is a cholic acid conjugate of the GR antagonist RU486 (12) that selectively acts in the liver to antagonize the GR. In leptin-deficient *ob/ob* mice, A-348441 successfully improved glucose homeostasis, without elevated circulating corticosterone (13). Using euglycemic-hyperinsulinemic clamps, it was demonstrated to decrease hepatic glucose output and increased insulin sensitivity in *fa/fa* rats, a genetic model of obesity (13, 14), making it a suitable model for our study.

## Research Methods and Design

Chemicals were from Sigma-Aldrich (Poole, UK) and primers from Invitrogen Life Technologies (Paisley, UK), unless otherwise stated. All molecular biology reagents and kits were from Qiagen (West Sussex, UK). Dutasteride was from AK Scientific (Union City, CA), and A-348441 was a gift from KaroBio (Huddinge, Sweden). The diets were from Research Diets, Inc. (New Brunswick, NJ), who also prepared the custom high-fat diets containing dutasteride and A-348441.

### Animals

Male mice deficient in 5*α*R1 (5*α*R1KO; n = 8 to 11 per group housed 2 to 4 mice per cage at 21°C) were bred in house by mating heterozygous mice with a mixed C57Bl6/SvEv/129 background (3, 4), with the corresponding mixed-strain wild-type littermates used as controls. The C57BL6/J male mice were from Harlan (Bicester, UK). The mice were studied under UK Home Office license and had free access to drinking water and food. The mice were culled (8:00 to 10:30 am) by decapitation with the trunk blood collected.

### Insulin signaling analysis

The insulin signaling pathways were studied in a subset of C57BL/6 mice. These were fasted for 4 hours (from 10:00 am to 2:00 pm) in clean cages, and human insulin (0.75 U/kg body weight in saline) was given via intraperitoneal injection. These mice were culled via decapitation after 15 minutes. In all cases, the tissues were dissected, wet weighed, and snap frozen on dry ice or fixed in 10% formalin.

### GR antagonism in 5*α*R1KO mice

Male 5*α*R1KO and corresponding littermate control mice (n = 8 to 11 per group; age, 12 weeks) were randomly allocated to a chow diet (11% fat, 0% sucrose; D12328); a Western style high-fat, high-sucrose diet (HFD; 58% kcal fat, 13% kcal sucrose; D12331) alone, or HFD containing A-348441 (120 mg/kg/d). After 9 weeks of experimental diet, the mice were fasted for 6 hours (8:00 am to 2:00 pm) before undergoing a glucose tolerance test (GTT; intraperitoneal injection; 2 mg/g), with blood taken by tail venesection at 0, 15, 30, 60, and 90 minutes. One week later, blood was taken at 8:00 am for basal corticosterone measurement, and 2 days later (after 10 weeks of the experimental diet), the mice were culled as stated. A-348441 was mixed within the HFD to achieve long-term administration without causing chronic stress that would result from repeat injection or gavage. This was especially desirable because disruption of 5*α*R1-mediated corticosterone clearance impairs the corticosterone stress response (15).

### GR antagonism after pharmacological inhibition of 5*α*Rs with dutasteride in C57BL/6 mice

C57BL6/J male mice (n = 8 to 12 per group; age, 12 weeks) were randomly allocated to chow diet, HFD alone, HFD containing A-348441 (120 mg/kg/d), HFD plus dutasteride (1.8 mg/kg/d), or HFD plus dutasteride plus A-348441 (same doses) for 4 weeks. GTTs were performed after 3 weeks of the experimental diet. The mice were culled 1 week later (after 4 weeks of the experimental diet).

### Laboratory analyses

#### Biochemistry

Plasma hormones were measured by ELISA as follows: corticosterone [Enzo Life Sciences, Exeter, UK; RRID: AB_2307314 (16)], insulin [Crystal Chem, Elk Grove Village, IL; RRID: AB_2783626 (17)], glucose by hexokinase assay (ThermoFisher, Loughborough, UK), triglycerides (ThermoFisher, Hemel Hempstead, UK), and nonesterified fatty acids (NEFAs; Zen-Bio, Research Triangle Park, NC) spectrophotometrically. Liver triglycerides were quantified as reported previously (18).

#### Quantification of mRNAs by real-time quantitative PCR

RNA was extracted as previously described (4). mRNA transcript abundances were quantified using real-time PCR, as previously described, with primers and probes as detailed by Livingstone *et al.* (4), McInnes *et al.* (19), and Nixon *et al.* (20). Transcript abundances for genes of interest were normalized for the mean abundance of the reference genes, *Tbp*, *Hprt*, and *Actβ*, the expression of which did not differ between groups.

#### Western blot analysis

Snap-frozen liver (∼40 μg) was homogenized with a rotor blade in ice-cold radioimmunoprecipitation assay buffer (Santa Cruz Biotechnology, Dallas, TX). Western blots were performed as previously described (19). The specific primary antibodies used were as follows: monoclonal rabbit anti-IR*β* [RRID: AB_2280448 (21)], polyclonal rabbit anti-phosphorylated AKT (Ser473) [RRID: AB_329825 (22)], polyclonal rabbit anti-AKT [RRID: AB_329827 (23)], polyclonal rabbit anti-phosphorylated GSK-3*β* (Ser9) [RRID: AB_331405 (24)], and monoclonal rabbit anti-GSK-3*β* [RRID: AB_490890 (25)], all from Cell Signaling Technologies (Leiden, Netherlands); polyclonal anti-rabbit GR [RRID: AB_2155784 (26)] from Santa Cruz Biotechnology, Inc. (Heidelberg, Germany); and monoclonal mouse anti-*β*-tubulin [RRID: AB_94650 (27)], monoclonal mouse anti-IRS1 [RRID: AB_11214300 (28)], and monoclonal mouse anti-IRS2 [RRID: AB_10615782 (29)], all from Millipore, Ltd. (Watford, UK). The secondary antibodies were IRDye 680RD goat anti-rabbit IgG (H+L) [RRID: AB_10956166 (30)], IRDye 800CW goat anti-rabbit IgG (H+L) [RRID: AB_621843 (31)], and IRDye 800CW goat anti-mouse IgG [RRID: AB_2782997 (32)], all from LI-COR Biosciences, Ltd. (Cambridge, UK). Phosphorylated antibodies were diluted 1:500 in BSA (Cohn Fraction V; 5% w/v in Tris-buffered saline-Tween), and all total protein primary antibodies were diluted 1:1000 in BSA (5% w/v in Tris-buffered saline-Tween). Secondary antibodies were diluted 1:10,000 in BSA when studying phosphorylation of proteins or milk when studying total protein. Band intensities were quantified using the Odyssey Imaging System (LI-COR Biosciences, Cambridgeshire, UK). Quantification of the proteins of interest was calculated by dividing their integrated intensity by that of the loading control (*β*-tubulin). Phosphorylated proteins were normalized to their corresponding bands of *β*-tubulin, and a ratio of (phosphorylated protein/*β*-tubulin)/(total protein/*β*-tubulin) was used in final analysis.

### Statistical analysis

Data are presented as the mean ± SEM, analyzed using GraphPad Prism, version 6 (San Diego, CA), using the Student *t* test or two-way ANOVA, with the Sidak post hoc test, as appropriate. Statistical significance was considered present at *P* < 0.05.

## Results

### Hepatic GR antagonism in mice with genetic disruption of 5*α*R1

Ten weeks of the HFD was sufficient to induce an adverse metabolic phenotype in the wild-type mice of mixed strain (wild-type HFD) background, causing excess weight gain of ∼4 g compared with that of the mice consuming the chow diet (9.0 ± 1.1 g vs 4.8 ± 0.5 g; *P* < 0.05). Furthermore, fasting insulin was increased by the HFD (2.8 ± 0.4 vs 2.0 ± 0.3 ng/mL; *P* < 0.05) without increasing the fasting glucose (HFD vs chow diet, 156.7 ± 3.7 mg/dL vs 151.6 ± 9.9 mg/dL). The liver triglyceride levels were also increased by the HFD (152.3 ± 18.4 vs 36.97 ± 9.4 μmol/g). The effects of A-348441 to attenuate the metabolic changes induced by 10 weeks of the HFD fat diet were thus studied in 5*α*R1KO (5*α*R1KO HFD) and wild-type HFD mice (the drug was not administered to the mice receiving chow diet). Consistent with a lack of central effects and previous data (12–14), the basal circulating corticosterone was not altered in either genotype after administration of A-348441 (Table 1).

**Table 1.** Indexes of Metabolism in Wild-Type and 5*α*R1KO Mice

Variable	Wild-Type		KO		*P* Value		
	HFD	HFD + A-348441	HFD	HFD A-348441	Effect of A-348441	Effect of Genotype	Interaction
Total weight gain, g	8.99 ± 1.11	5.04 ± 0.62*^a^*	8.83 ± 1.37	7.61 ± 0.55	< 0.01	0.21	0.16
Tissue, % body weight							
Omental adipose	0.062 ± 0.008	0.048 ± 0.005	0.079 ± 0.009	0.062 ± 0.007	0.05	0.06	0.85
Liver	4.52 ± 0.16	4.38 ± 0.12	4.55 ± 0.12	4.83 ± 0.08	0.59	0.07	0.12
Gonadal adipose	3.91 ± 0.32	3.33 ± 0.21	4.14 ± 0.36	3.83 ± 0.13	0.11	0.19	0.61
Perinephric adipose	1.30 ± 0.11	1.11 ± 0.08	1.42 ± 0.11	1.38 ± 0.08	0.24	0.06	0.47
Mesenteric adipose	1.60 ± 0.10	1.28 ± 0.07	1.59 ± 0.22	1.59 ± 0.10	0.23	0.24	0.21
Subcutaneous adipose	2.08 ± 0.26	1.77 ± 0.12	2.54 ± 0.34	2.01 ± 0.18	0.08	0.14	0.65
Quadriceps muscle	1.14 ± 0.06	1.19 ± 0.03	1.05 ± 0.05	1.23 ± 0.02	0.20	0.10	0.76
Interscapular adipose	0.51 ± 0.03	0.54 ± 0.05	0.52 ± 0.03	0.53 ± 0.06	0.36	0.05	0.64
Biochemistry							
Corticosterone, nM	35.2 ± 2.2	37.9 ± 2.1	35.9 ± 3.5	38.5 ± 3.3	0.10	0.45	0.82
Plasma TGA, mmol/L	2.07 ± 0.20	1.79 ± 0.16	2.01 ± 0.20	1.87 ± 0.07	0.14	0.80	0.92
Fasting NEFA, mM	1.58 ± 0.12	1.91 ± 0.14	1.26 ± 0.08	1.38 ± 0.10	0.06	< 0.01	0.40
*Δ*NEFA, mM (0–15 min of GTT)	0.29 ± 0.16	0.44 ± 0.10	0.06 ± 0.11	0.38 ± 0.07	0.04	0.15	0.33

Mice were fed the HFD or HFD plus A-348441 diet for 10 wk.

Data presented as mean ± SEM and compared using two-way ANOVA with Sidak *post hoc* tests.

Abbreviation: TGA, triglycerides.

^a^ Codes of comparisons (*P* < 0.05) within same genotype: vs HFD, n = 8 to 11 per group.

**Figure 1.**
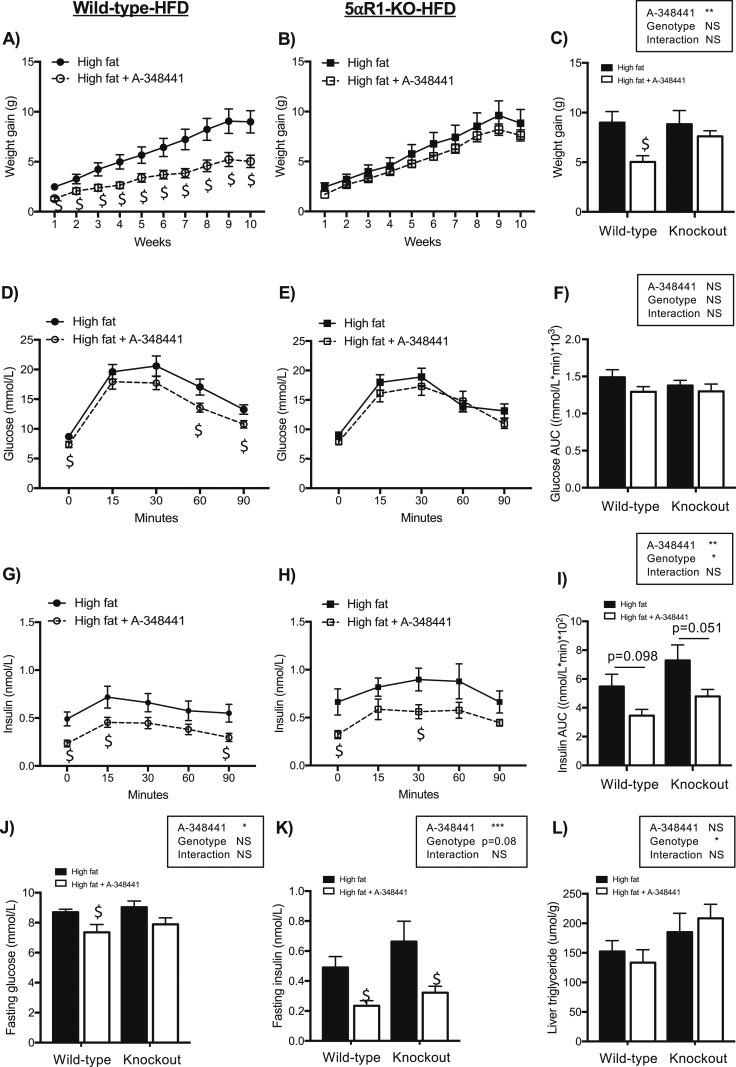
Body weight gain and glucose homeostasis in 5*α*R1-deficient (KO) mice and their wild-type controls after an HFD and administration of A-348441 for 10 wk. Mice were fed an HFD or HFD plus A-348441. A-348441 ameliorated the increase in body weight gain in the (A) wild-type but not (B, C) KO mice. (D–F) A-348441 did not significantly affect glucose levels in either genotype in response to GTTs but (G–I) had an overall effect of ameliorating the increase in insulin levels (*P* = 0.004). After 9 wk of experimental diet, (J) A-348441 had lowered the fasting glucose in the wild-type mice only but (K) had ameliorated the increase in fasting insulin in both genotypes. KO mice overall had (I) higher insulin and (L) hepatic triglyceride levels than the wild-type mice. Data are presented as the mean ± SEM and by the individual genotype for clarity, with analysis of all data by two-way ANOVA followed by the Sidak *post hoc* test (^$^*P* < 0.05 vs matched genotype receiving the HFD; n = 8 to 11 per group). For overall ANOVA: *P < 0.05; **P < 0.01; ***P < 0.001. NS, not significant.

The weight gain induced by the HFD was attenuated by A-348441 in the wild-type HFD mice but not in the 5*α*R1KO HFD mice (Fig. 1A–1C). However, the mass of specific adipose depots was not decreased by the drug or different by genotype (Table 1). A-348441 lowered the fasting glucose in the wild-type HFD mice only (Fig. 1D, 1E, and 1J) but had no effect on the overall glucose response to a GTT (Fig. 1F). An overall effect of A-348441 was found to lower the fasting insulin level (Fig. 1G, 1H, and 1K) and the insulin response to a GTT (Fig. 1I) in mice of both genotypes. Similar to a previous report (4), 5*α*R1KO HFD mice were predisposed to develop insulin resistance, with a trend toward a higher fasting insulin level (Fig. 1K) and an increased insulin response to a glucose challenge compared with wild-type HFD mice, irrespective of the presence of A-348331 (Fig. 1I). Surprisingly, the 5*α*R1KO HFD mice overall had lower fasting NEFAs compared with the wild-type HFD mice (Table 1). As a measure of adipose insulin sensitivity, NEFA suppression during the first 15 minutes of the GTT was measured. However, A-348441 did not influence NEFA suppression but increased fasting NEFAs in the wild-type HFD mice only (Table 1). Furthermore, as anticipated, 5*α*R1KO HFD mice, overall, had higher hepatic triglyceride levels than did their wild-type controls; however, A-348441 did not lower hepatic or plasma triglyceride levels in either genotype (Fig. 1L; Table 1).

#### Liver-selective GR antagonism after dutasteride administration in C57BL/6 mice

**Figure 2.**
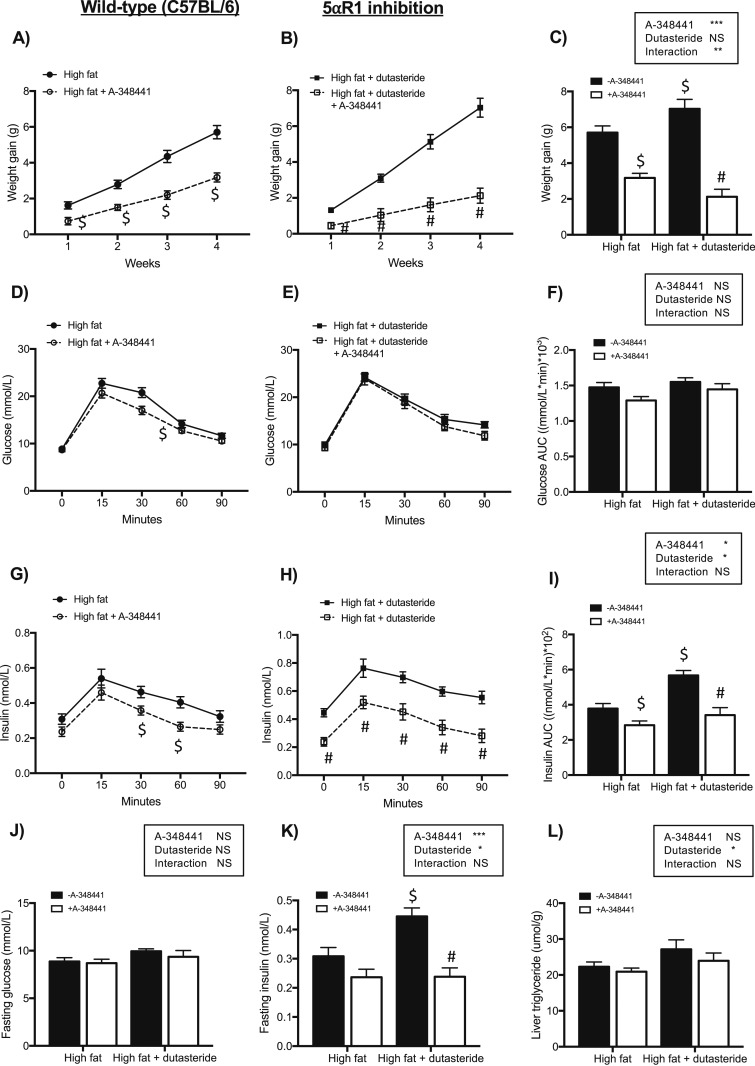
Body weight and glucose homeostasis in C57BL/6 mice after dutasteride and A-348441 administration for 4 wk. C57BL/6 mice were fed an HFD, HFD plus A-348441, HFD plus dutasteride, or HFD plus dutasteride plus A-348441 diet. A-348441 ameliorated the increased body weight in both (A) mice fed the HFD alone and (B) mice fed the HFD plus dutasteride. (C) Mice consuming the HFD plus dutasteride had increased body weight gain vs the mice consuming the HFD alone. (D–F) Glucose tolerance was not changed by dutasteride or A-348441 administration. A-348441 lowered the increased insulin levels induced by (G) the HFD alone and (H) in combination with dutasteride during a GTT. (I) Dutasteride increased the insulin levels compared with mice consuming the HFD alone. A-348441 (J) had no effect on fasting glucose but (K) normalized the increased fasting insulin caused by dutasteride combined with the HFD. (L) With a HFD, an overall effect was found for dutasteride to increase hepatic triglyceride levels; however, A-348441 did not ameliorate this increase. Data are presented as the mean ± SEM and stratified by 5*α*R status for clarity, with analysis of all data using two-way ANOVA at each point, followed by the Sidak *post hoc* test (^$^*P* < 0.05 vs HFD; ^#^*P* < 0.05 vs HFD plus dutasteride; n = 8 to 12 per group; NS, not significant). For overall ANOVA: *P < 0.05; **P < 0.01; ***P < 0.001.

Mice with a C57BL/6 background are predisposed to diet-induced obesity and were studied for a shorter period. After only 4 weeks, a high-fat diet induced weight gain in excess of that in the chow-fed mice (6.3 ± 0.4 vs 4.0 ± 0.1 g; *P* < 0.05), accompanied by a doubling in fasting insulin of 2.1 ± 0.2 vs 0.8 ± 0.1 ng/mL (*P* < 0.05) and an increase in hepatic triglyceride content (22.3 ± 1.3 μmol/g vs 10.5 ± 1.7 μmol/g; *P* < 0.05). Similar to the littermate controls of the 5*α*R1KO mice (Fig. 1), C57BL/6 mice on A-348441 had reduced weight gain (Fig. 2A) and no changes in glucose tolerance (Fig. 2D) but lowered insulin levels (Fig. 2G).

Successful administration of dutasteride was confirmed by a reduction in prostate size (Table 2). Coadministration of dutasteride with the HFD exacerbated the adverse metabolic phenotype in C57BL/6. The body weight gain had increased after 4 weeks compared with the HFD alone (Fig. 2C), with increased adipose weight gain in the omental depot only (Table 2). Dutasteride increased the insulin response to a bolus of glucose during a GTT compared with the HFD alone (Fig. 2I), as well as fasting insulin (Fig. 2K), without altering the glucose response to GTT or fasting glucose (Fig. 2F and 2J). Compared with mice receiving the HFD alone, mice fed dutasteride had increased hepatic triglyceride levels (Fig. 2L), without differences in plasma triglycerides, fasting plasma NEFAs, or suppression of NEFAs after the glucose challenge (Table 2). Food intake was not measured per mouse but on a cage basis (with limited power of n = 3), appeared to be less in the mice fed A-348441, irrespective of 5*α*R status (16.9 ± 0.8 vs 13.5 ± 0.7 kCal per mouse daily).

**Table 2.** Indexes of Metabolism in C57BL/6 Mice Fed HFD, With or Without A-348441 and/or Without Dutasteride

Variable	HFD	HFD + A-348441	HFD + Dutasteride	HFD + Dutasteride + A-348841	*P* Value		
					Effect of Dutasteride	Effect of A-348441	Interaction
Total weight gain, g	5.71 ± 0.37	3.18 ± 0.25^*a*^	7.03 ± 0.52^*a*^	2.13 ± 0.42^*b*^	0.73	< 0.001	0.005
Tissue weight, % body weight							
Omental adipose	0.049 ± 0.003	0.036 ± 0.003	0.069 ± 0.006^*a*^	0.043 ± 0.008^*b*^	0.010	< 0.001	0.195
Liver	4.89 ± 0.08	4.71 ± 0.12	5.16 ± 0.12	4.56 ± 0.28	0.704	0.016	0.187
Gonadal adipose	3.28 ± 0.17	2.32 ± 0.19^*a*^	3.46 ± 0.17	2.07 ± 0.18	0.838	< 0.001	0.249
Perinephric adipose	0.99 ± 0.05	0.56 ± 0.06^*a*^	0.99 ± 0.06	0.51 ± 0.07	0.726	< 0.001	0646
Mesenteric adipose	1.46 ± 0.09	0.97 ± 0.07^*a*^	1.56 ± 0.09	1.04 ± 0.05	0.354	< 0.001	0.844
Subcutaneous adipose	1.53 ± 0.09	1.02 ± 0.06^*a*^	1.85 ± 0.13	1.37 ± 0.15^*b*^	0.004	< 0.001	0.934
Quadriceps muscle	1.24 ± 0.03	1.24 ± 0.04	1.17 ± 0.03	1.29 ± 0.03	0.734	0.098	0.092
Interscapular adipose	0.38 ± 0.03	0.42 ± 0.03	0.44 ± 0.03	0.32 ± 0.02^*b*^	0.507	0.184	0.013
Prostate^*c*^	0.049 ± 0.005	NC	0.024 ± 0.004^*a*^	0.034 ± 0.003^*a*^	NA	NA	NA
Biochemistry							
Corticosterone, nM	86.23 ± 10.9	106.6 ± 23.4	32.69 ± 3.9^*a*^	88.34 ± 22.8	0.035	0.027	0.29
Plasma TGA, mmol/L	2.03 ± 0.14	1.56 ± 0.13^*a*^	2.07 ± 0.12	1.76 ± 0.13	0.37	0.008	0.57
Fasting NEFA, mM	2.13 ± 0.11	1.98 ± 0.15	2.08 ± 0.01	1.54 ± 0.15^*b*^	0.15	0.048	0.25
*Δ*NEFA, mM (0–15 min of GTT)	1.11 ± 0.11	0.98 ± 0.16	1.36 ± 0.15	0.89 ± 0.13	0.61	0.08	0.31

Mice were fed the HFD, HFD + A-348441, HFD + dutasteride, or HFD + dutasteride + A-348441 for 4 wk.

Data presented as mean ± SEM and compared using two-way ANOVA with Sidak *post hoc* tests.

Abbreviations: NA, not applicable; NC, not collected; TGA, triglycerides.

^a^ *P* < 0.05 vs HFD alone.

^b^ *P* < 0.05 vs HFD plus dutasteride.

^c^ Prostate weight was compared using one-way ANOVA: *P* = 0.002; n = 8 to 12 per group.

In contrast with genetic deletion of 5*α*R1, coadministration of A-348441 with dutasteride reduced the excess weight gain (Fig. 2B), in parallel with reduced expansion of visceral adipose depots and lower fasting NEFAs (Table 2). Coadministration of A-348441 normalized the increased fasting insulin and insulin response to GTT caused by dutasteride (Fig. 2I and 2K), again without affecting the fasting glucose or glucose response during a GTT (Fig. 2E and 2J). Just as in the 5*α*R1KO mice, coadministration of A-348441 did not abrogate the increased liver triglyceride levels resulting from dutasteride (Fig. 2L) nor alter NEFA suppression (Table 2).

#### Effects of A-348441 on glucocorticoid and bile acid-sensitive hepatic pathways after administration to C57BL/6 mice

**Figure 3.**
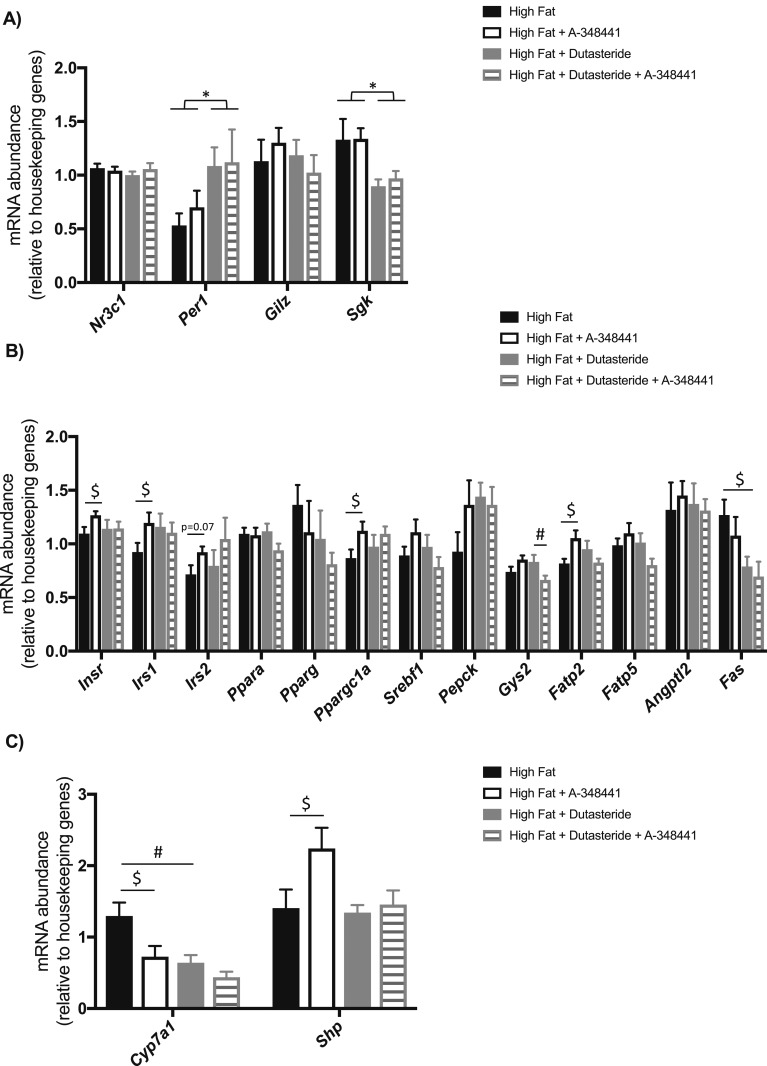
Response of hepatic transcripts to A-348441 in C57BL/6 mice after 4 wk of experimental diet. mRNA abundance was quantified for *Nr3c1*, *Per1, Gilz, Sgk, Insr*, *Irs1*, *Irs2*, *Ppara*, *Pparg*, *Ppargc1a*, *Srebf1, Pepck, Gys2, Fatp2, Fatp5, Angpt12, Fas, Cyp7a1*, and *Shp.* (A) Among glucocorticoid-sensitive genes, dutasteride increased *Per1* and lowered *Sgk*, although A-348441 had no effect. Dutasteride also lowered *Fas* mRNA, and A-348441 increased transcript abundance for *Insr*, *Irs1*, and *Ppargc1a*. (B) When administered in combination with dutasteride, A-348441 lowered *Gys2* transcript levels. Both dutasteride and A-348441 lowered *Cyp7a1* abundance, but (C) only A-348441 alone increased *Shp* transcript levels compared with the HFD. Data are presented as mean ± SEM and analyzed using two-way ANOVA, followed by Sidak *post hoc* test (^$^*P* < 0.05 vs HFD; ^#^*P* < 0.05 vs HFD plus dutasteride). For overall ANOVA: *P < 0.05 for effect of dutasteride, n = 8 (HFD plus dutasteride plus A-348441); n = 10 (HFD plus dutasteride); n = 12 (HFD and HFD plus A-348441) per group.

As expected, dutasteride treatment increased the expression of glucocorticoid-sensitive genes such as *Per1* in the liver (Fig. 3A). It also resulted in reduced expression of the *Sgk* transcript, although *Gilz* was unaffected. Liver-selective GR antagonism did not alter expression of any of these glucocorticoid-sensitive transcripts (Fig. 3A). Neither dutasteride nor A-348441 affected the hepatic abundance of *Nr3c1* mRNA (Fig. 3A).

The transcript abundance of genes involved in bile acid signaling was also investigated. A-348441 lowered *Cyp7a1* mRNA abundance and increased *Shp* abundance compared with the HFD (Fig. 3C). Dutasteride alone also lowered *Cyp7a1* mRNA abundance but not *Shp*. A-348441 administered in combination with dutasteride had no further effect on *Cyp7a1* transcript abundance compared with dutasteride administration alone.

### Hepatic insulin signaling was unaffected by A-348441 administration

**Figure 4.**
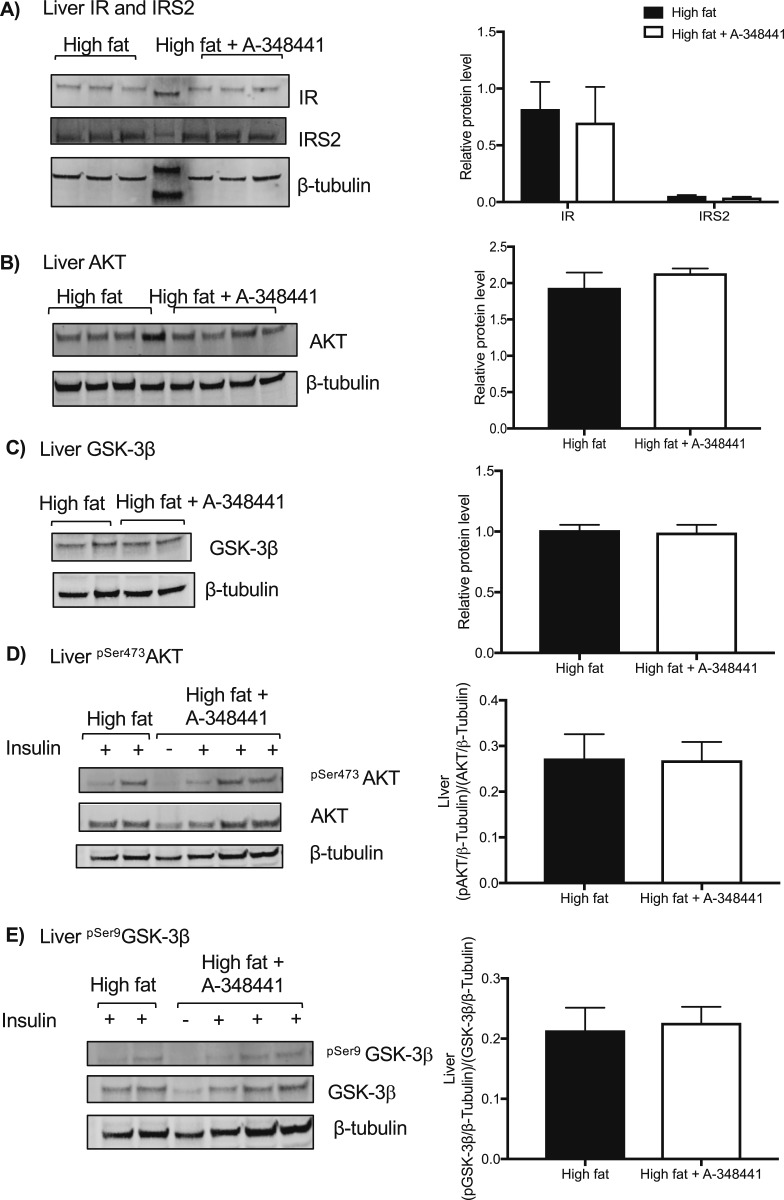
Response of hepatic insulin signaling proteins to A-348441 in C57BL/6 mice. Total protein levels of (A) hepatic IR, IRS2, (B) AKT, and (C) GSK-3*β* did not change in response to administration of A-348441. Phosphorylation status of (D) hepatic ^pSer473^AKT and (E) hepatic ^pSer9^GSK-3*β* after insulin stimulation did not change in response to the addition of A-348441. Data are presented as mean ± SEM and were analyzed using the Student *t* test. *P < 0.05 vs HFD, n = 6 (HFD); n = 12 (HFD plus A-348441) per group.

A-348441 did not alter the total protein levels of IR, IRS2, AKT, or GSK-3*β* in the liver (Fig. 4A–4C). A-348441 lowered *Gys2* transcript levels only in the presence of dutasteride. Although dutasteride decreased *Fas* mRNA levels, it had little effect on other hepatic metabolic transcripts (Fig. 3B). A-348441 treatment increased the transcript abundance of *Insr*, *Irs1*, *Ppargc1a*, and *Fatp2*, with a trend (*P* = 0.07) toward greater *Irs2* mRNA transcripts (Fig. 3B). When administered in combination with dutasteride, A-348441 lowered the transcript abundance of *Gys2* only.

To better assess insulin signaling, we gave a subset of mice in each of the treatment groups a bolus of insulin after a 4-hour fast and collected tissue 15 minutes later. However, no changes were observed in pSer473AKT and pSer9 GSK-3*β* signaling in the liver (Fig. 4D and 4E).

## Discussion

Deficiency or inhibition of 5*α*R1 predisposes men and mice to insulin resistance and fatty liver (4, 7–9). However, because 5*α*R1 can metabolize multiple steroids, notably both glucocorticoids and androgens, it has been unclear which hormone plays the more crucial role in the phenotype. Rodents with deficiency in 5*α*R1 developed hyperinsulinemia and hepatic steatosis even after androgen levels were decreased through castration (4), implicating glucocorticoids as the key hormone involved. Because 5*α*R1 is highly expressed in the liver, an organ crucial to metabolic balance, and glucocorticoid levels in this tissue are approximately doubled in 5*α*R1KO mice (4), we hypothesized that the predisposition to insulin resistance and fatty liver after 5*α*R1 deficiency was due to an increase in glucocorticoid action in the liver. Using A-348441, a liver-selective hepatic GR antagonist, we demonstrated in two models of 5*α*R1 deficiency, 5*α*R1KO mice and administration of dutasteride (a dual 5*α*-reductase inhibitor), that glucocorticoids contribute to the phenotype.

To test this hypothesis, we initially used a genetic approach using 5*α*R1KO mice. These are bred from a mixed genetic background (3), and the differences in metabolic parameters between 5*α*R1KO mice and their littermate controls develop slowly with consumption of a high-fat diet (4, 9). The intervention with A-348441 was begun at 12 weeks of age for 10 weeks during the decline in metabolic health. At this point, 5*α*R1KO mice consuming a high-fat diet had increased insulin levels and a greater insulin response during GTT compared with their littermate controls. However, as expected, differences in weight were not yet observed [this aspect of the phenotype requires 6 months to manifest (4, 9)]. The predisposition to fatty liver in 5*α*R1KO mice compared with their littermate controls was also already evident during this shorter period and before overt changes in body weight between the genotypes was observed.

The outcome of treatment with A-348441 in the wild-type mice was similar to that observed in the *fa/fa* rats (13, 14), with improved insulin sensitivity and attenuation of weight gain. However, hepatic steatosis was unresponsive to hepatic GR antagonism. Previously, genetic disruption of hepatic GR in a *db/db* background had attenuated hepatic triglyceride accumulation (33). Likewise, amplification of hepatic generation of glucocorticoids by overexpression of 11*β*-hydroxysteroid dehydrogenase 1 exacerbated steatosis (34). However, the lack of change is in agreement with previous work from Macfarlane *et al.* (35). They showed that systemic glucocorticoid blockade with RU486 did not ameliorate excess liver fat in patients with type 2 diabetes mellitus (35). The beneficial metabolic changes resulting from A-348441 were less marked in the 5*α*R1KO mice than in their littermate controls. Although administration of A-348441 attenuated the excess weight gain induced by the HFD in the littermate control mice, this was unaffected in the 5*α*R1KO mice. In terms of glucose disposal, A-348441 reversed the hyperinsulinemia to a similar extent between the 5*α*R1KO mice and their littermate controls. Again, hepatic steatosis caused by the HFD was unresponsive to intervention, and the 5*α*R1KO mice maintained higher liver fat levels than their littermate controls with both diets. This was supported by the fact that A-348441 did not lower the transcript abundance of *Fatp2* nor *Fatp5* in the liver.

Given that the overall phenotype of the 5*α*R1KO was improved but remained different from that of the wild-type mice, these observations suggest that GR activation plays a role in HFD-induced metabolic dysfunction on this mixed genetic background but do not support the hypothesis that it is solely glucocorticoid mediated, unless insufficient drug had been administered, which we believe unlikely. Had excess GR activation been solely responsible, the difference between genotypes would have been negated. Equivalent doses of RU486 to the dose of A-348441 used in our study and common in mouse models would be expected to achieve concentrations in the blood well in excess of those of corticosterone (∼100 nM) and, hence, unlikely to be limiting in the liver (36, 37).

To gain further insight, a hepatic-selective GR antagonism was pursued in a second model, that of pharmacological inhibition with dutasteride, to better reflect the clinical therapeutic scenario. The 5*α*R1KO mice have a life-long deficiency in steroid metabolism, including during development, and might have undergone compensatory changes in steroid hormone action and metabolism. In a clinical setting, 5*α*R inhibition generally starts later in middle-age adults. Dutasteride, a dual 5*α*R inhibitor, was administered to C57BL/6 mice, a strain predisposed to become obese, hyperglycemic, and hyperinsulinemic owing to a naturally occurring deletion in nicotinamide nucleotide transhydrogenase (38). The C57BL/6 mice were glucose intolerant and hyperinsulinemic after only 3 weeks of a HFD; thus, this shorter period for the study was chosen, matching the insulin increment in wild-type mice in the previous experiment. The ability of 5*α*R inhibition to adversely affect metabolism was apparent after 4 weeks of treatment, reminiscent of studies in humans (4, 7). Specifically, dutasteride exacerbated the weight gain, increased the fasting insulin and insulin response to a GTT, and induced hepatic steatosis in mice fed a high-fat diet (4).

Just as with the wild-type controls of 5*α*R1KO mice, the HFD-fed C57BL/6 mice treated with A-348441 alone demonstrated an improved metabolic phenotype. The exacerbation of metabolic dysfunction by dutasteride was also prevented by A-348441, highlighting a substantial role of hepatic glucocorticoids. The weight gain was normalized to that of the chow-fed mice, and the insulin response to a glucose challenge and plasma triglycerides levels were all decreased. Moreover, the white adipose depots were reduced in weight. Systemic benefits might have been caused by hemocrine effects of hepatic metabolic mediators, instead of direct effects from the liver-selective drug. Other studies have demonstrated manipulation of metabolic signals in the liver such as FOXO1 and IR, as well as PI3K activity, can indirectly affect the adipose tissue mass and systemic insulin sensitivity (39). However, hepatic steatosis was again not affected by hepatic GR antagonism, reinforcing the suggestion of a different etiology. Previous studies have shown excess steatosis induced by pharmacological inhibition of 5*α*Rs was also not reversed by castration, which suggests androgens alone are also not the sole cause (4). Either a complex interplay between glucocorticoids and androgens underpins the liver steatosis (40) or alternative hormonal pathways are involved. Nevertheless, we found most aspects of the observed adverse metabolic phenotype were responsive to GR blockade, suggesting that excess hepatic glucocorticoid action plays a substantial role in mediating many of the metabolic effects of dutasteride. To further confirm the contribution of increased hepatic glucocorticoid levels to changes in insulin sensitivity and hepatic steatosis, dutasteride could be administrated to liver-specific GR knockout mice consuming a high-fat diet.

To gain insight into the potential mechanism responding to hepatic GR antagonism, hepatic transcript abundance of the genes involved in insulin signaling and the lipid metabolism were studied. The level of *Nr3c1* (GR) mRNA was unaffected by the drug. *Per1* mRNA was studied as a known GR-responsive transcript. The levels of *Per1* were elevated by dutasteride but were not suppressed after GR antagonism. Very few changes in hepatic transcripts that might explain the phenotypic changes were seen in response to GR antagonism, with only some limited increase in genes associated with insulin signaling such as *Insr, Irs1*, and *Ppargc1a* and a small reduction in *Gys2*. However, a similar lack of regulation of genes involved in gluconeogenesis or lipid metabolism was observed when hepatic glucocorticoid ligand availability was suppressed in mice with liver-selective disruption of 11*β*-hydroxysteroid dehydrogenase 1 (41). Protein expression from the insulin signaling pathway was also explored under insulin stimulation; however, A-348441 had no effect on the total or phosphorylated state of these proteins in the liver (42, 43). Further studies using insulin clamps would permit greater insight, and a greater difference might be observed if the tissues were collected after a longer duration of the HFD and A-348441.

Given that hemocrine mechanisms might underpin the improved phenotype, it is important to consider the tissue selectivity of the antagonist. The drug had been developed to attenuate hepatic GR stimulation. Predosing with A-348441 antagonized glucocorticoid-induced upregulation of hepatic tyrosine aminotransferase activity by 79% and suppressed glucocorticoid-induced hepatic glycogen formation by 59% (13). Furthermore, compared with RU486, a systemic GR antagonist, A-348441 had substantially less effects in nonhepatic tissues (*e.g.,* it was 12-fold less potent in preventing glucocorticoid-induced upregulation of glutamine synthetase in L6 skeletal muscle myocytes and 15-fold less potent in a glucocorticoid-stimulated model of adipocyte differentiation in 3T3-L1 cells) (13). This highly liver-selective profile avoided adverse effects on the hypothalamic pituitary adrenal axis incurred through systemic actions or central GR antagonism. In line with previous studies, we were able to recapitulate that A-348441 did not affect the circulating concentrations of corticosterone, suggesting that negative feedback via GR within the brain was unaffected by the presence of an antagonist, suggesting minimal circulating levels (13).

GR is the most likely target for the drug; however, because it is a conjugate of RU486 and cholic acid, A-348441 is hepato-selective through the presence of a bile acid. Bile acids can modulate insulin signaling pathways via nuclear hormone receptors such as farnesoid X receptor-*α* (FXR*α*) and GR (44, 45). It was thought unlikely that A-348441 would have off-target effects on the bile acid pathway, because the Kd for binding of cholic acid to FXR*α* is exceedingly high (36 mM vs a Kd of 0.27 nM for A-348441 to GR) (12) and not anticipated with the given doses. However, we found that A-348441 lowered *Cyp7a1* and increased *Shp* mRNA abundance. Nonetheless, it is likely that the effects of GR and FXR*α* are linked—hepatic GR can regulate systemic bile acid homeostasis, with glucocorticoids inhibiting the transcriptional activity of FXR (45, 46). Furthermore, SHP is a corepressor of the glucocorticoid receptor (47); increased mRNA of *Shp* would still have the end result of decreased GR action. *Cyp7a1* and *Shp* mRNA abundance in mice fed dutasteride was not significantly different from that of the mice given dutasteride plus A-348441. Altogether, A-348441 might influence hepatic bile acid signaling; however, the effects of A-348441 in ameliorating the adverse phenotype caused by dutasteride were not through the same pathways. Had A-348441 lowered androgen levels and activation of the androgen receptor, this would have confounded our interpretation. However, we found no evidence of this (*e.g.,* a lack of reduction in prostate weight).

In conclusion, most, but not all, of the adverse metabolic effects of diet-induced obesity brought about by inhibition of 5*α*R1 with dutasteride can be reversed by liver-selective GR antagonism. Our data support a substantial role for increased hepatic GR activation in the adverse metabolic phenotype observed after pharmacological inhibition and a contributory role after genetic disruption of 5*α*R1. However, the signaling pathways underpinning the changes were elusive. A-348441 proved a useful tool to dissect the role of liver GR in regulating the metabolism and has revealed that many of the consequences of a high-fat diet in animal models, with the crucial exception of steatosis, can be reversed by targeting hepatic GR. The results of the present study have consolidated the hypothesis that men receiving dual 5*α*R inhibitors are exposed to challenges to their metabolic health. Concomitant administration of therapies that attenuate glucocorticoid action, such as 11*β*-hydroxysteroid dehydrogenase 1 inhibitors (48, 49) might prove to be of benefit.

## Data Availability

The datasets generated during and/or analyzed during the current study are not publicly available but are available from the corresponding author on reasonable request.

## Abbreviations:

5*α*R: 5*α*-reductase
5*α*R1: 5*α*-reductase type 1
5*α*R1-KO: 5*α*-reductase 1 knockout
5*α*R2: 5*α*-reductase type 2
FXR: farnesoid X receptor
GR: glucocorticoid receptor
GTT: glucose tolerance test
HFD: Western-style high-fat, high-sucrose diet
NEFA: nonesterified fatty acid
